# Morphological and Phylogenetic Analyses Reveal Three New Species of *Didymella* (*Didymellaceae, Pleosporales*) from Jiangxi, China

**DOI:** 10.3390/jof10010075

**Published:** 2024-01-18

**Authors:** Xingxing Luo, Yafen Hu, Jiwen Xia, Kai Zhang, Liguo Ma, Zhaohuan Xu, Jian Ma

**Affiliations:** 1 College of Agronomy, Jiangxi Agricultural University, Nanchang 330045, China; luoxingxing99@126.com (X.L.); hyf604325418@126.com (Y.H.); hzzhaohuan@163.com (Z.X.); 2 Shandong Provincial Key Laboratory for Biology of Vegetable Diseases and Insect Pests, College of Plant Protection, Shandong Agricultural University, Taian 271018, China; xiajiwen1@126.com; 3 College of Forestry Engineering, Shandong Agriculture and Engineering University, Jinan 250100, China; kaise0907@126.com; 4 Shandong Key Laboratory of Plant Virology, Institute of Plant Protection, Shandong Academy of Agricultural Sciences, Jinan 250100, China; maliguo809@163.com

**Keywords:** asexual ascomycetes, *Dothideomycetes*, multi-locus phylogeny, new taxa, taxonomy

## Abstract

*Didymella* contains numerous plant pathogenic and saprobic species associated with a wide range of hosts. Over the course of our mycological surveys of plant pathogens from terrestrial plants in Jiangxi Province, China, eight strains isolated from diseased leaves of four host genera represented three new species of *Didymella*, *D. bischofiae* sp. nov., *D. clerodendri* sp. nov., and *D. pittospori* sp. nov. Phylogenetic analyses of combined ITS, LSU, *RPB2,* and *TUB2* sequence data, using maximum-likelihood (ML) and Bayesian inference (BI), revealed their taxonomic placement within *Didymella*. Both morphological examinations and molecular phylogenetic analyses supported *D. bischofiae*, *D. clerodendri,* and *D. pittospori* as three new taxa within *Didymella*. Illustrations and descriptions of these three taxa were provided, along with comparisons with closely related taxa in the genus.

## 1. Introduction

*Didymella*, the type genus of the family *Didymellaceae*, was introduced by Saccardo in 1880, with *D. exigua* as the type species, and later validated when a Latin diagnosis was provided [1,2]. The genus was recently emended by Chen et al. [3,4], who gave a very detailed account of generic concepts. The sexual morphs of *Didymella* are mainly characterized by solitary or confluent, ostiolate pseudothecial ascomata with multi-layered, pseudoparenchymatous ascomatal walls and cylindrical to clavate or saccate, 8-spored, bitunicate asci with hyaline or brownish uniseptate (symmetrical or asymmetrical) or multiseptate ascospores. The asexual morphs of *Didymella* are mainly characterized by solitary or confluent, ostiolate or poroid, pycnidial conidiomata with multi-layered, pseudoparenchymatous conidiomatal walls, and phialidic, hyaline conidiogenous cells that produce smooth conidia, which are generally aseptate, variable in shape, hyaline or occasionally pigmented, and larger or septate in at least one species in older cultures. Unicellular chlamydospores are often present in pure culture [2,3,4,5,6,7]. To date, 438 records of *Didymella* are listed in *Species Fungorum* [8], and most of them are usually found as saprobes from herbaceous and woody plants, but many are also important plant pathogens [3,4,9].

*Didymella* is an old, species-rich genus, but its early taxonomic placements are uncertain. The genus was originally described in the family *Mycosphaerellaceae* and later placed in *Pleosporaceae*, *Phaeosphaeriaceae*, *Venturiaceae*, or *Pleosporales* genera *incertae sedis* [2,4]. De Gruyter et al. [2] introduced a new family *Didymellaceae* with *Didymella* as the type genus to accommodate *Ascochyta*, *Didymella*, *Phoma,* and several related *phoma*-like genera based on evidence from phylogenetic analyses of combined LSU and SSU sequence data. Aveskamp et al. [5] indicated that *Didymella* appears to be polyphyletic, with some members mixed with other taxa of *Leptosphaerulina*, *Macroventuria*, *Microsphaeropsis*, *Peyronellaea*, and suggested that *Didymella* is in urgent need of taxonomic revision. Chen et al. [3] further clarified the generic delimitation in *Didymellaceae* using a morpho-molecular approach; *Didymella* was restricted to a monophyletic group and encompassed 37 species. Since then, 49 further species were added based on morphological and phylogenetic analyses [4,7,9,10,11,12,13,14,15,16,17,18,19,20,21,22,23,24,25,26], but *D. acutilobae*, *D. erhaiensis*, *D. gongkaensis*, *D. hippuris,* and *D. myriophyllana* were considered invalid species under the ICN Art. 40.8 or Art. F.5.1 [27].

Jiangxi Province is located on the south bank of the middle and lower reaches of the Yangtze Riverin southern China. It lies at 24°29′–30°04′ N and 113°34′–118°28′ E and covers a total area of 166,900 km^2^ with superior ecological environment, humid subtropical climate, and abundant plant resources, which provide favorable conditions for the survival and multiplication of various microbial species. During an investigation of the diversity of plant pathogens from terrestrial plants in Jiangxi Province, three interesting species of *Didymella* were collected from the symptomatic leaves of four host genera. Based on morphological and multi-loci (LSU, ITS, *RPB2,* and *TUB2*) phylogenetic analyses, they are proposed as new to science in the present study, and their names were registered in *Index Fungorum* [28].

## 2. Materials and Methods

### 2.1. Sample Collection and Fungal Isolation

Samples of diseased leaves were collected from botanical garden or conservation areas with rich plant resources in Jiangxi Province, China. Representative plants samples with leaf spots were placed in Ziploc™ bags, labeled, and returned to the laboratory. The strains from the collected diseased leaves were isolated and identified using a tissue separation method [29]. Before isolation, the collected leaf samples were rinsed with running water, and several tissue pieces (5 mm × 5 mm) from the junction of diseased and healthy parts were selected for surface disinfection. The tissue pieces were disinfected with 75% ethanol for 45 s and 5% sodium hypochlorite for 30 s, rinsed 3 times with sterile water, dried with sterilized filter paper, transferred to the potato dextrose agar (PDA; 20% potato + 2% dextrose + 2% agar, *w*/*v*) plates [30], and then incubated at 25 °C in darkness for 3–5 days. The growing hyphae at the edge of the colony was inoculated onto new PDA plates for purification and morphological studies.

### 2.2. Morphological and Cultural Characterization

Each fungal isolate was removed to the new PDA, MEA, and OA plates and incubated at 25 °C in darkness. Their morphological characters were recorded after 7 days. Morphological characteristics of conidia on PDA were observed using an Olympus BX 53 light microscope and captured using the Olympus DP 27 digital camera (Olympus Optical Co., Tokyo, Japan) with a 40 × objective at the same background color and scale, and the sizes of conidia were randomly selected for measurement. All fungal strains were stored in 10% sterilized glycerin at 4 °C for further studies. The studied specimens and cultures were deposited in the Herbarium of Jiangxi Agricultural University, Plant Pathology, Nanchang, China (HJAUP).

### 2.3. DNA Extraction, PCR Amplification, and Sequencing

Fungal isolates were incubated on PDA plates at 25 °C for 7–14 days. The hyphae were scraped from the surface of colonies and transferred into 2 mL microcentrifuge tubes for genomic DNA extraction. DNA extraction was carried out using the Solarbio Fungi Genomic DNA Extraction Kit (Beijing Solarbio Science & Technology Co., Ltd., Beijing, China). To confirm the species, the regions (ITS, LSU, *RPB2,* and *TUB2*) of all fungal isolates were sequenced. A portion of the internal transcribed spacer region (ITS), large ribosomal subunit (LSU), β-tubulin (*TUB2*) regions, and the second largest subunit of RNA polymerase II (*RPB2*) genes were amplified using primer pairs ITS5/ITS4 [31], LR0R/LR7 [32], Bt2a/Bt2b [33], and dRPB2-5f/dRPB2-7r [34], respectively. The corresponding primer pairs and PCR processes are listed in Table 1. The total volume of the PCR reaction was 20 μL, including 10 μL of 2 × Power Taq PCR Master Mix, 0.8 μL of each the forward and reverse primer, 7.4 μL of double-distilled water (ddH2O), and 1 μL of DNA template. The PCR products were visualized on a 1% agarose gel electrophoresis and stained with ethidium bromide. Sequencing was performed bidirectionally by Beijing Tsingke Biotechnology Co., Ltd. (Beijing, China). Newly obtained sequences in this study were deposited in NCBI GenBank (www.ncbi.nlm.nih.gov, accessed on 21 November 2023; Table 2).

### 2.4. Phylogenetic Analyses

The newly generated sequences from this study were analyzed using other related sequences obtained from GenBank (Table 2). Sequences of the individual loci were initially aligned using MAFFTv.7 [35] on the online server (http://maffTh.cbrc.jp/alignment/server/, accessed on 18 December 2023) using default settings and manually corrected where necessary. Phylogenetic analyses were first conducted individually for each locus, and then for a combined analyses of four loci (ITS, LSU, *TUB2,* and *RPB2*). The ITS, LSU, *RPB2*, and *TUB2* sequence data were concatenated by using the “Concatenate Sequence” function in Phylosuite software v1.2.1 [36], and absent sequences data in the comparisons were treated using the question mark and “–” as missing data. The concatenated aligned dataset was analyzed separately using maximum-likelihood (ML) and Bayesian inference (BI). The best evolutionary model for each alignment dataset was selected using ModelFinder [37] and incorporated into the analyses. Maximum-likelihood phylogenies were inferred using IQ-TREE [38] under an edge-linked partition model for 10,000 ultrafast bootstraps [39]. The optima trees were inferred using the heuristic search option with 1000 random sequence additions. The best-fit model was TIM2e+I+G4 for ITS, LSU, *TUB2,* and *RPB2* alignments. Based on the partition model (2 parallel runs, 2,000,000 generations), Bayesian inference phylogenies were inferred using MrBayes 3.2.6 [40], in which the initial 25% of sampled data was discarded as burn-in, and the best nucleotide substitution model for each locus was identified using ModelFinder of Phylosuite to be SYM+I+G4 for ITS and GTR+F+I+G4 for LSU, *RPB2,* and *TUB2*. The resulting trees were plotted using FigTree v.1.4.2 [36] (http://tree.bio.ed.ac.uk/software/figtree, accessed on 18 December 2023) and further edited in Adobe Illustrator 2021.

## 3. Results

### 3.1. Molecular Phylogeny

**Figure 1:**
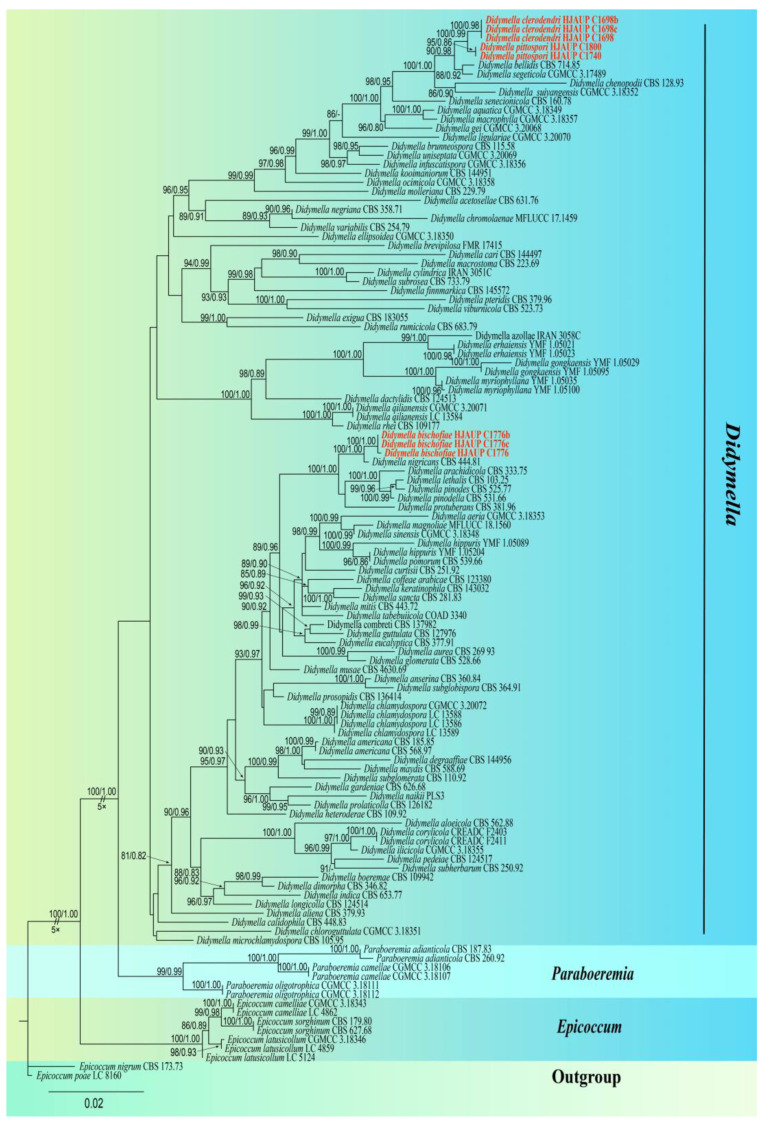
Phylogram of *Didymellaceae* based on concatenated ITS, LSU, *TUB2,* and *RPB2* sequence data. Significant MLBS/BPP support values above 80% and 0.80 are given at the nodes. The tree is rooted to *Epicoccum nigrum* (CBS 173.73) and *E. poae* (LC 8160). Strains from the present study are indicated in red. Two branches were shortened according to the indicated multipliers to fit the page size, and these are indicated by the symbol (//).

Based on the sequence data of ITS, LSU, *RPB2,* and *TUB2*, the phylogenetic relationships of the eight strains of *Didymella* were analyzed using the regions of four genes of 118 strains representing 96 species in *Didymellaceae*. The combined data set (ITS:1–462, LSU:463–1189, *RPB2*:1190–1630, and *TUB2*:1631–1915) was composed of 477 distinct patterns, 341 parsimony informative sites, 61 singleton sites, and 1513 constant sites. A total of four single-locus data sets, ITS, LSU, *RPB2,* and *TUB2*, contained 54, 19, 171, and 97 parsimony informative sites, respectively. *Epicoccum nigrum* (CBS 173.73) and *E. poae* (LC 8160) served as outgroups. The phylogenetic reconstructions obtained from the combined dataset of maximum-likelihood and Bayesian inference analyses support largely similar topologies, and the best-scoring ML consensus tree (lnL = –14164.265) is shown in Figure 1. The maximum-likelihood bootstrap support (MLBS) values above 80% and Bayesian posterior probability (BPP) greater than 0.80 are shown in the first and second position above the nodes. Our eight strains nested within the genus *Didymella* representing three new phylogenetic species, *D. bischofiae*, *D. clerodendri,* and *D. pittospori*. The strain of *D. bischofiae* (HJAUP C1776, HJAUP C1776b, and HJAUP C1776c) forms a distinct clade sister to *D. nigricans* (CBS 444.81) with strong statistical support (MLBS/BPP = 100/1.00); *D. clerodendri* (HJAUP C1698, HJAUP C1698band HJAUP C1698c) forms a high-support clade (MLBS/BPP = 100/0.99) with *D. pittospori* (HJAUP C1740 and HJAUP C1800), and they form a sister clade to *D. bellidis* (CBS 714.85) and *D. segeticola* (CGMCC 3.17489), with strong statistical support (MLBS/BPP = 90/0.98).

### 3.2. Taxonomy

**Figure 2:**
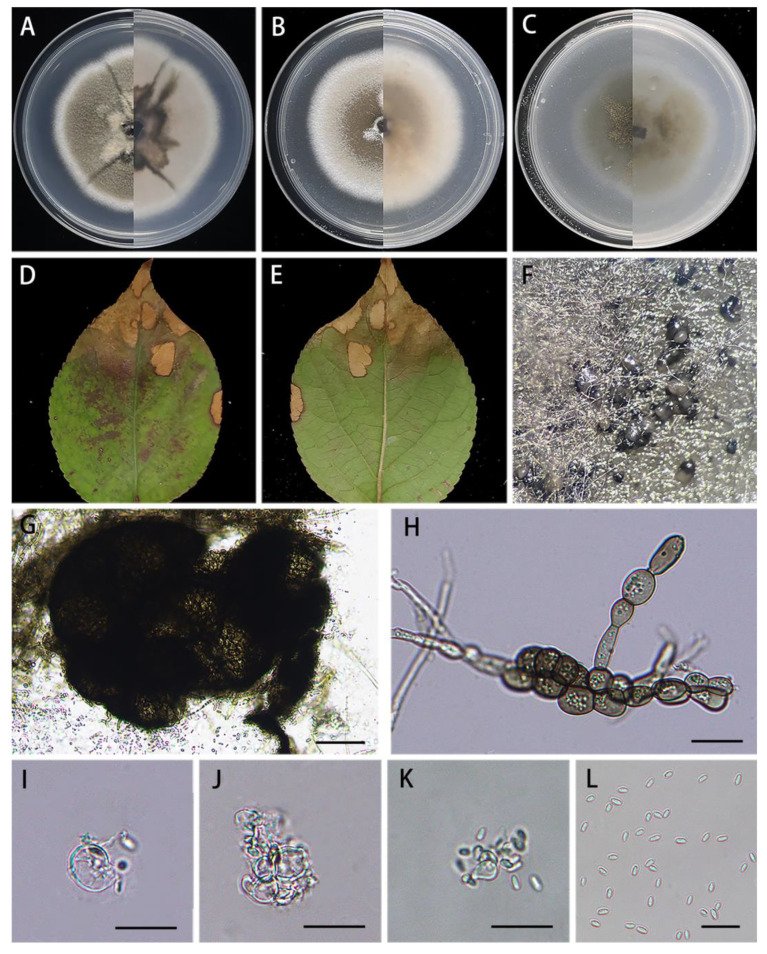
*Didymella bischofiae* (HJAUP M1776, holotype): (**A**). Colony after 7 days on PDA (front and reverse); (**B**). Colony after 7 days on MEA (front and reverse); (**C**). Colony after 7 days on OA (front and reverse); (**D**,**E**)**.** Leaf of host plant (front and reverse); (**F**)**.** Pycnidia forming on PDA; (**G**)**.** Pycnidium; (**H**)**.** Chlamydospores; (**I**–**K**). Conidiogenous cells; (**L**)**.** Conidia. Scale bars: (**G**) = 50 μm; (**H**–**L**) = 20 μm.

*Didymella bischofiae* X.X. Luo, X.G. Zhang, and Jian Ma, sp. nov., Figure 2.

Index Fungorum number: IF901249.

Etymology: Referring to the host genus from which it was collected, *Bischofia polycarpa*.

Holotype: HJAUP M1776.

Description: Irregular leaf spots, yellow–brown in center, and pale red halos at margin. Asexual morph on PDA: *Conidiomata* are pycnidial, superficial, solitary or aggregated, subglobose, black, ostiolate, 195–292 × 131–232 μm (*n* = 20), with 1–2 papillate ostioles. *Conidiogenous cells* are phialidic, hyaline, smooth, ampulliform, 5.4–10.1 × 4.6–8.1 μm (*n* = 15). *Conidia* are ovoid or ellipsoidal, hyaline, smooth, thin-walled, aseptate, 4.1–7.1 × 1.9–3.1 μm ($\overset{-}{x}$ = 5.5 × 2.4 μm, *n* = 40), mostly without guttules. *Conidial matrix* are pale white. Sexual morph not observed.

Culture characteristics: Colonies on PDA reaching 68–70 mm diam after 7 days at 25 °C, margin regular, aerial mycelium sparsely, flat, central pale olivaceous and white all around, reverse slightly pink, and abundant production of chlamydospores with growth. Colonies on MEA reaching 62–63 mm diam after 7 days at 25 °C, margin regular, the middle is pale brown and gradually becomes white around, covered with medium aerial mycelium, and reverse buff to white. Colonies on OA reaching 58–60 mm diam after 7 days at 25 °C, margin regular, pale olivaceous, and reverse concolorous.

Material examined: Xinyu Subtropical Forest Park, Jiangxi Province, China, on diseased leaves of *Bischofia polycarpa* (H.Lév.) Airy Shaw (*Euphorbiaceae*), 2 November 2022, X.X. Luo, HJAUP M1776 (holotype), ex-type living culture HJAUP C1776.

Notes: Strains HJAUP 1776, HJAUP 1776b, and HJAUP 1776c are similar in morphological characteristics and have identical DNA sequences; form a single, high support clade (MLBS/BPP = 100/1.00, Figure 1); and, therefore, are identified as the same new species, *Didymella bischofiae*. The phylogenetic tree showed that the strains of *D. bischofiae* formed a distinct lineage sister to *D. nigricans* (CBS 444.81) in a fully supported clade (MLBS/BPP = 100/1.00, Figure 1). *Didymella bischofiae* is closely related to *D. nigricans* and has 8 bp differences in four loci from the latter. Morphologically, *D. bischofiae* clearly differed from *D. nigricans* which produce fewer chlamydospores, smaller conidiogenous cells (4–8 × 5–8 μm vs. 5.4–10.1 × 4.6–8.1 μm), and allantoid to subcylindrical conidia mostly with 2–3 guttules [5,41].

**Figure 3:**
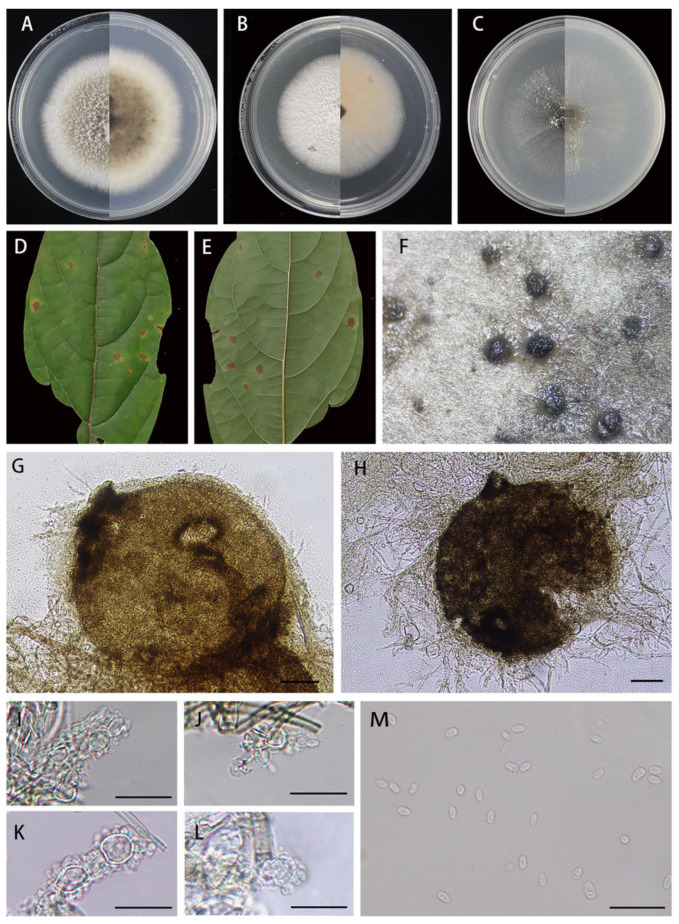
*Didymella clerodendri* (HJAUP M1698, holotype): (**A**)**.** Colony after 7 days on PDA (front and reverse); (**B**). Colony after 7 days on MEA (front and reverse); (**C**). Colony after 7 days on OA (front and reverse); (**D**,**E**). Leaf of host plant (front and reverse); (**F**)**.** Pycnidia forming on PDA; (**G**,**H**). Pycnidium; (**I**–**L**). Conidiogenous cells; (**M**)**.** Conidia. Scale bars: (**G**,**H**) = 50μm; (**I**–**M**) = 20 μm.

*Didymella clerodendri* X.X. Luo, X.G. Zhang, and Jian Ma, sp. nov., Figure 3.

Index Fungorum number: IF901250.

Etymology: Referring to the host genus from which it was collected, *Clerodendrum cyrtophyllum*.

Holotype: HJAUP M1698.

Description: Irregular leaf spots, brown in center, and yellow to yellowish at margin. Asexual morph on PDA: *Conidiomata* are pycnidial, superficial, solitary or aggregated, mostly globose or subglobose, darker brown, with hyphal out growths, ostiolate, 206–330 × 190–290 μm (*n* = 20). Ostioles are single, central, and slightly papillate. *Conidiogenous cells* are hyaline, smooth, phialidic, subglobose, ampulliform to lageniform, 6.2–9.9 × 3.9–6.9 μm (*n* = 15). *Conidia* are ovoid or ellipsoidal, hyaline, smooth, thin walled, aseptate, 4.3–5.7 × 2.0–3.0 μm ($\overset{-}{x}$ = 5.0 × 2.5 μm, *n* = 40), mostly with one or two minutes guttules. *Conidial exudates* buff. Sexual morph not observed.

Culture characteristics: Colonies on PDA reaching 68–70 mm diam after 7 days at 25 °C, margin regular, light brown in the middle, covered by white felt-like aerial hyphae, and white around; abundant production of pycnidia in the late growth stage. Colonies on MEA reaching 62–65 mm diam after 7 days at 25 °C, margin regular, covered by white felt-like aerial hyphae, and the back was buff. Colonies on OA reaching 57–59 mm diam after 7 days at 25 °C, margin regular, pale olivaceous, covered by a small amount of whitish aerial hyphae, and reverse pale olivaceous.

Material examined: Jingdezhen National Forest Park, Jiangxi Province, China, on diseased leaves of *Clerodendrum cyrtophyllum* Turcz. (*Lamiaceae*), 2 November 2022, X.X. Luo, HJAUP M1698 (holotype), ex-type living culture HJAUP C1698.

Notes: Strains HJAUP 1698, HJAUP 1698b, and HJAUP 1698c are similar in morphological characteristics and have identical DNA sequences; form a single, high support clade (MLBS/BPP = 100/0.98, Figure 1); and, therefore, are identified as the same new species, *Didymella clerodendri*. The phylogenetic tree showed that *D. clerodendri* clustered with *D. pittospori* in a high supported clade (MLBS/BPP = 100/0.99, Figure 1), and they form a sister clade to *D. bellidis* (CBS 714.85) and *D. segeticola* (CGMCC 3.17489) with a well-supported clade (MLBS/BPP = 90/0.98, Figure 1). *Didymella clerodendri*is closely related to *D. bellidis*, *D. segeticola,* and *D. pittospori*, and has, respectively, 26 bp, 22 bp, and 20 bp differences from *D. bellidis*, *D. segeticola,* and *D. pittospori* in four loci. Moreover, *D. clerodendri* morphologically differs from *D. pittospori* in having smaller pycnidia (206–330 × 190–290 μm vs. 290–496 × 151–323 μm), larger conidiogenous cells (6.2–9.9 × 3.9–6.9 μm vs. 4.7–7.2 × 3.6–5.6 μm), and larger conidia (4.3–5.7 × 2.0–3.0 μm vs. 3.1–5.2 × 1.6–2.5 μm). *Didymella clerodendri* is also morphologically similar to *D. segeticola*, but the latter has smaller pycnidia (90–105 × 75–95 μm vs. 206–330 × 190–290 μm), smaller conidiogenous cells (5–6.5 × 4–5.5 μm vs. 6.2–9.9 × 3.9–6.9 μm), and larger conidia (4.5–7 × 2.5–4 μm vs. 4.3–5.7 × 2.0–3.0 μm) with 1–6 polar guttules [12,42].

**Figure 4:**
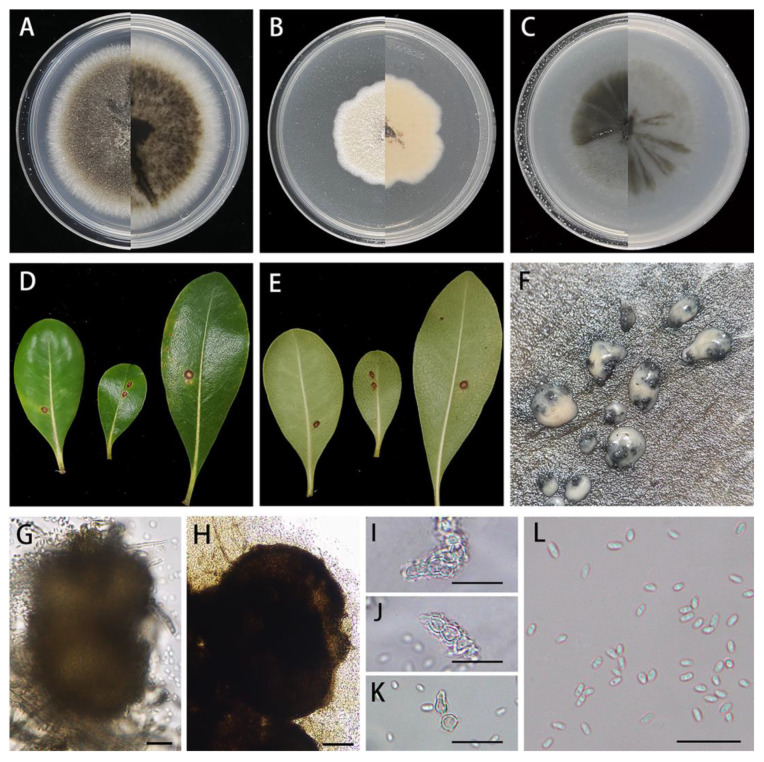
*Didymella pittospori* (HJAUP M1740, holotype): (**A**). Colony after 7 days on PDA (front and reverse); (**B**). Colony after 7 days on MEA (front and reverse); (**C**). Colony after 7 days on OA (front and reverse); (**D**,**E**). Leaf of host plant (front and reverse); (**F**). Pycnidia forming on PDA; (**G**,**H**). Pycnidium; (**I**–**K**). Conidiogenous cells; (**L**). Conidia. Scale bars: (**G**,**H**) = 50μm; (**I**–**L**) = 20 μm.

*Didymella pittospori* X.X. Luo, X.G. Zhang, and Jian Ma, sp. nov., Figure 4.

Index Fungorum number: IF901251.

Etymology: Referring to the host genus from which it was collected, *Pittosporum tobira*.

Holotype: HJAUP M1740.

Description: Round leaf spots, brown to light-brown in center, and tan to black at margins with yellowish halos. Asexual morph on PDA: *Conidiomata* are pycnidial, superficial, solitary, solitary or aggregated, mostly globose or subglobose, black, glabrous, ostiolate, 290–496 × 151–323 μm (*n* = 20). Ostioles are single and slightly papillate. *Conidiogenous cells* are phialidic, hyaline, smooth, ampulliform to lageniform, 4.7–7.2 × 3.6–5.6 μm (*n* = 15). *Conidia* are oblong, ovoid or ellipsoidal, hyaline, smooth, thin-walled, aseptate, 3.1–5.2 × 1.6–2.5 μm ($\overset{-}{x}$ = 4.1 × 2.0 μm, *n* = 40), and mostly with two minutes guttules. *Conidial matrix* is milky white. Sexual morph not observed.

Culture characteristics: Colonies on PDA reaching 74–76 mm diam after 7 days at 25 °C, margin regular, aerial mycelium sparsely, flat, dark brown near the central zone and white around, and reverse concolorous; abundant production of pycnidia in the late growth stage. Colonies on MEA reaching 46–50 mm diam after 7 days at 25 °C, margin waved, light-brown in the middle, white around, covered by white felt-like aerial hyphae, and reverse buff. Colonies on OA reaching 62–65 mm diam after 7 days at 25 °C, margin regular, olivaceous, covered by a few whitish aerial hyphae, and reverse concolorous.

Material examined: Longhu Mountain Nature Reserve, Jiangxi Province, China, on diseased leaves of *Pittosporum tobira* W.T.Aiton (*Pittosporaceae*), 3 November 2022, X.X. Luo, HJAUP M1740 (holotype), ex-type living culture HJAUP C1740; Jingdezhen Botanical Garden, Jiangxi Province, China, on diseased leaves of *Eriobotrya japonica* (Thunb.) Lindl. (*Rosaceae*), 2 November 2022, X.X. Luo, HJAUP M1800 (paratype), ex-paratype living culture HJAUP C1800.

Notes: Strains HJAUP 1740 and HJAUP 1800 are similar in morphological characteristics and have identical DNA sequences; form a single, strong support clade (MLBS/BPP = 95/0.86, Figure 1); and, therefore, are identified as the same new species, *Didymella pittospori*. The phylogenetic tree showed that *D. pittospori* clustered with *D. clerodendri* in a well-supported clade (MLBS/BPP = 100/0.99, Figure 1). *Didymella pittospori* is closely related to *D. clerodendri* and has 20 bp differences from *D. clerodendri* in four loci. Moreover, *D. pittospori* is morphologically distinguished from *D. clerodendri* as having larger pycnidia (290–496 × 151–323 μm vs. 206–330 × 190–290 μm), smaller conidiogenous cell (4.7–7.2 × 3.6–5.6 μm vs. 6.2–9.9 × 3.9–6.9 μm), and smaller conidia (3.1–5.2 × 1.6–2.5 μm vs. 4.3–5.7 × 2.0–3.0 μm). *Didymella pittospori* is also different from *D. segeticola*, which has smaller pycnidia (90–105 × 75–95 μm vs. 290–496 × 151–323 μm) and bigger conidia (4.5–7 × 2.5–4 μm vs. 3.1–5.2 × 1.6–2.5 μm) with 1–6 polar guttules [12,42].

## 4. Discussion

There are many kinds of fungi in Jiangxi Province, and the fungal groups are complex. Relevant studies have shown that several mycological investigations are also constantly exploring and enriching the fungal diversity (e.g., [12,43,44,45,46]). In this study, we isolated plant pathogens from diseased leaves of a wide range of plant hosts in Jiangxi Province, China. Based on the morphomolecular approach, three new species of *Didymella*, *D. bischofiae*, *D. clerodendri,* and *D. pittospori,* were introduced, which contributed to the supplementation of the diversity of this genus.

The establishment of *Didymella* was based on morphological studies. To date, there are 438 records for *Didymella* in *Species Fungorum* [8] but most of them are identified only by morphology, and only 86 species (including five invalid species) have sequence data so far. Morphological characteristics are significant for the identification of fungi, but the research based on morphological characteristics alone is not comprehensive. With the increase inavailable sequences for *Dothideomycetes* species, the molecular phylogenetic analysis has helped to clarify the phylogenetic relationships among the members of *Dothideomycetes*, and further clarified the species boundaries for the *Didymella* via multilocus analyses. However, studies conducted on *Didymella* have no universally accepted standards in selecting barcodes for phylogenetic analyses. For instance, De Gruyter et al. [2] established the family *Didymellaceae* with *Didymella* as type genus, but the initial *Didymella* species had only SSU and LSU sequences. Woudenberg et al. [47] and Thambugala et al. [48] introduced *D. clematidis* and *D. eriobotryae* using ITS, LSU, and *TUB2*. Liu et al. [49] introduced *D. cirsii* using ITS and LSU. Chen et al. [3,12] introduced 47 *Didymella* species using ITS, LSU, *TUB2,* and *RPB2*. Crous et al. [15,16] introduced *D*. *cari* and *D. finnmarkica* using ITS, LSU, *ACT,* and *TUB2* or *RPB2*. From 2020 onwards, except for *D. nakii*, *D. azollae,* and *D. brevipilosa* using ITS, LSU, and *TUB2* or*RPB2*, the further described 27 *Didymella* species were introduced using ITS, LSU, *RPB2,* and *TUB2* [4,7,9,10,11,13,17,19,20,21,22,24,25,26]. Recent studies indicated that the use of LSU, ITS, *TUB2,* and *RPB2* shows good phylogenetic resolution in revealing the phylogeny of *Didymella* and related genera within *Didymellaceae*. However, BLASTn analyses of these sequences showed that ITS and LSU sequences in some *Didymella* species have a high similarity, whereas *RPB2* and/or *TUB2* have distinct nucleotide differences. For example, ITS, LSU, *RPB2,* and *TUB2* of *D. degraaffiae* [24] (MN823444, MN823295, MN824470, and MN824618) were 99.36, 99.72, 95.13, and 91.89% similar to *D. maydis* [3] (FJ427086, EU754192, GU371782, and FJ427190); *D. qilianensis* [4] (MT229701, MT229678, MT239098, and MT249269) were 100, 100, 98.32, and 98.2% similar to *D. rhei* [3] (GU237743, GU238139, KP330428, and GU237653). Our new species, *D. clerodendri* (OR625709, OR625714, OR620207, and OR611942) were 99.81, 100, 98.82, and 99.68% similar to *D. pittospori* (OR905550, OR905581, OR947922, and OR934715). Considering this phenomenon, we found that ITS and LSU sequences maybe show important significance in resolving the phylogeny of *Didymellaceae*, whereas *RPB2* and/or *TUB2* significantly increase the phylogenetic resolution in distinguishing *Didymella* species. Other loci in the mitochondrial genomemay also provide important insights in resolving the phylogeny of fungi [50,51,52], but hitherto not a single mitogenome exists for *Didymella* species.

To date, studies conducted on *Didymella* have mainly focused on their alpha-taxonomy, and most species are considered to be saprobes or phytopathogens of woody and herbaceous hosts [4,9,13,28,42], whereas only a few species have been isolated from inorganic substrates, such as *D. glomerata* and *D. pomorum* from inorganic materials including asbestos, cement, paint, etc. [3,12,53]. Recent studies also show that four *didymella* species, *D. gardeniae*, *D. heteroderae*, *D. musae,* and *D. microchlamydospora,* were found from human nail or cornea lesion [23], but there is no relevant data that support whether it has a direct relationship with the human disease. The genus *Didymella* is mainly recorded from China, Germany, India, Italy, The Netherlands, New Zealand, South Africa, and USA [3,4,7,12,21]; little published information is recorded in other regions [28]. Scant attention has been accorded to the roles of their ecosystem function, substrate specificities, and fungal pathogenicity. Thus, the understanding of external factors that affect fungal lifestyles may have a significant impact on agricultural development, ecological environment, and human health, contributing significantly to the field of plant pathology and fungal taxonomy.

## Figures and Tables

**Table 1:** Loci used in this study with the corresponding PCR primers and conditions.

Locus	Primers	Sequence 5′-3′	PCR Program
ITS	ITS5	GGAAGTAAAAGTCGTAACAAGG	94 °C: 3 min, (94 °C: 15 s, 55 °C: 15 s, 72 °C: 30 s) ×35 cycles, 72 °C: 5 min
	ITS4	TCCTCCGCTTATTGATATGC	
*TUB2*	Bt2a	GGTAACCAAATCGGTGCTGCTTTC	94 °C: 3 min, (94 °C: 15 s, 55 °C: 15 s, 72 °C: 30 s) × 35 cycles, 72 °C: 5 min
	Bt2b	ACCCTCAGTGTAGTGACCCTTGGC	
LSU	LSU-LR0R	GTACCCGCTGAACTTAAGC	94 °C: 3 min, (94 °C: 15 s, 55 °C: 15 s, 72 °C: 30 s) × 35 cycles, 72 °C: 5 min
	LSU-LR7	TACTACCACCAAGATCT	
*RPB2*	dRPB2-5f	GAYACNGAYGAYCGWGAYCAYTTYGG	94 °C: 3 min, (94 °C: 15 s, 56 °C: 15 s, 72 °C: 2 min) × 35 cycles, 72 °C: 5 min
	dRPB2-7r	AANCCCATDGCYTGYTTDCCCAT	

**Table 2:** Species and GenBank accession numbers of the sequences used in the phylogenetic analyses. New sequences are in bold.

Species	Strain Number	Host, Substrate	Host Family	Locality	GenBank Accession Numbers			
					LSU	ITS	*RPB2*	*TUB2*
*Didymella acetosellae*	CBS 631.76	*Rumex acetosella*	*Polygonaceae*	UK	MN943749	MN973542	MT018176	MT005645
*D. aeria*	CGMCC 3.18353^T^ = LC 7441	Air		China	KY742205	KY742051	KY742137	KY742293
*D. aliena*	CBS 379.93 = PD 82/945	*Berberis* sp.	*Berberidaceae*	The Netherlands	GU238037	GU237851	KP330416	GU237578
*D. aloeicola*	CBS 562.88^T^	*Aloe* sp.	*Asphodelaceae*	Italy	MN943742	MN973535	MT018164	MT005638
*D. americana*	CBS 185.85 = PD 80/1191	*Zea mays*	*Poaceae*	USA	GU237990	FJ426972	KT389594	FJ427088
*D. americana*	CBS 568.97^T^	*Glycine max*	*Fabeceae*	USA	GU237991	FJ426974	MN983437	FJ427090
*D. anserina*	CBS 360.84	Potato flour		The Netherlands	GU237993	GU237839	KT389596	GU237551
*D. aquatica*	CGMCC 3.18349^T^ = LC 5556	Water		China	KY742209	KY742055	KY742140	KY742297
*D. arachidicola*	CBS 333.75^T^ = ATCC 28,333 = IMI 386,092 = PREM 44889	*Arachis hypogaea*	*Fabeceae*	South Africa	GU237996	GU237833	KT389598	GU237554
*D. aurea*	CBS 269.93^T^ = PD 78/1087	*Medicago polymorpha*	*Fabeceae*	New Zealand	GU237999	GU237818	KT389599	GU237557
*D. azollae*	IRAN 3058C^T^	*Azolla filiculoides*		Iran	MT514912	MT514915	–	MT512518
*D. bellidis*	CBS 714.85 = PD 74/265	*Bellis perennis*	*Asteraceae*	The Netherlands	GU238046	GU237904	KP330417	GU237586
***D. bischofiae***	**HJAUP C1776** ^T^	***Bischofia polycarpa***	***Euphorbiaceae***	**China**	**OR625713**	**OR625712**	**OR620208**	**OR620206**
***D. bischofiae***	**HJAUP C1776b**	***Bischofia polycarpa***	***Euphorbiaceae***	**China**	**OR905564**	**OR905553**	**–**	**OR934716**
***D. bischofiae***	**HJAUP C1776c**	***Bischofia polycarpa***	***Euphorbiaceae***	**China**	**OR905561**	**OR905554**	**–**	**OR934717**
*D. boeremae*	CBS 109942^T^ = PD 84/402	*Medicago littoralis* cv. Harbinger	*Fabeceae*	Australia	GU238048	FJ426982	KT389600	FJ427097
*D. brevipilosa*	FMR 17415; CBS 148654	Plant debris		Spain	OU612372	OU612373	OU612359	OU612358
*D. brunneospora*	CBS 115.58^T^ = DSM 62044	*Chrysanthemum roseum*	*Asteraceae*	Germany	KT389723	KT389505	KT389625	KT389802
*D. calidophila*	CBS 448.83^T^	Soil		Egypt	GU238052	FJ427059	MT018170	FJ427168
*D. cari*	CBS 144497^T^	*Coriandrum sativum*	*Apiaceae*	Canada	MH327861	MH327825	–	MH327899
*D. chenopodii*	CBS 128.93 = PD 79/140	*Chenopodium quinoa* cv. Sajana	*Chenopodiaceae*	Peru	GU238055	GU237775	KT389602	GU237591
*D. chlamydospora*	LC 13586	*Elymus glaucus*	*Poaceae*	China	MT229671	MT229694	MT239091	MT249262
*D. chlamydospora*	CGMCC 3.20072 = LC 13587^T^	*Elymus glaucus*	*Poaceae*	China	MT229672	MT229695	MT239092	MT249263
*D. chlamydospora*	LC 13588	*Polygonum viviparum*	*Polygonaceae*	China	MT229673	MT229696	MT239093	MT249264
*D. chlamydospora*	LC 13589	*Polygonum sibiricum*	*Polygonaceae*	China	MT229674	MT229697	MT239094	MT249265
*D. chloroguttulata*	CGMCC 3.18351^T^ = LC 7435	Air		China	KY742211	KY742057	KY742142	KY742299
*D. chromolaenae*	MFLUCC 17-1459^T^	*Chromolaena odorata*	*Asteraceae*	Thailand	MT214457	MT214363	–	–
***D. clerodendri***	**HJAUP C1698** ^T^	***Clerodendrum cyrtophyllum***	***Lamiaceae***	**China**	**OR625714**	**OR625709**	**OR620207**	**OR611942**
***D. clerodendri***	**HJAUP C1698b**	***Clerodendrum cyrtophyllum***	***Lamiaceae***	**China**	**OR905576**	**OR905545**	**OR947923**	**OR934711**
***D. clerodendri***	**HJAUP C1698c**	***Clerodendrum cyrtophyllum***	***Lamiaceae***	**China**	**OR905575**	**OR905546**	**OR947921**	**OR934712**
*D. coffeae-arabicae*	CBS 123380^T^ = PD 84/1013	*Coffea arabica*	*Rubiaceae*	Ethiopia	GU238005	FJ426993	KT389603	FJ427104
*D. combreti*	CBS 137982^T^	*Combretum mossambicensis*	*Combretaceae*	Zambia	KJ869191	KJ869134	MT018139	MT005626
*D. corylicola*	CBS 146357; CREADC-F2403^T^	*Corylus avellana*	*Betulaceae*	Italy	MN954290	MN954301	MN958323	MN958333
*D. corylicola*	CREADC-F2411	*Corylus avellana*	*Betulaceae*	Italy	MN954298	MN954309	MN958330	MN958340
*D. curtisii*	PD 86/1145 = CBS 251.92	*Nerine* sp.	*Amaryllidaceae*	The Netherlands	GU238013	FJ427038	MT018131	FJ427148
*D. cylindrica*	IRAN 3051C	*Pteridium aquilinum*	*Pteridiaceae*	Iran	OK257022	OK257014	OK247736	OK247741
*D. dactylidis*	PD 73/1414 = CBS 124513^T^	*Dactylis glomerata*	*Poaceae*	USA	GU238061	GU237766	MT018173	GU237599
*D. degraaffiae*	CBS 144956^T^	Soil		The Netherlands	MN823295	MN823444	MN824470	MN824618
*D. dimorpha*	CBS 346.82^T^	*Opuntia* sp.	*Cactaceae*	Spain	GU238068	GU237835	MT018158	GU237606
*D. ellipsoidea*	CGMCC 3.18350^T^ = LC 7434	Air		China	KY742214	KY742060	KY742145	KY742302
*D. erhaiensis*	YMF1.05023	*Hydrocharis dubia*	*Hydrocharitaceae*	China	MH257457	MH257369	MH311809	MH422997
*D. erhaiensis*	YMF1.05021^T^	*Eichhornia crassipes*	*Pontederiaceae*	China	MH257455	MH257367	MH311807	MH422995
*D. eucalyptica*	PD 79/210 = CBS 377.91	*Eucalyptus* sp.	*Myrtaceae*	Australia	GU238007	GU237846	KT389605	GU237562
*D. exigua*	CBS 183.55^T^	*Rumex arifolius*	*Polygonaceae*	France	EU754155	GU237794	EU874850	GU237525
*D. finnmarkica*	CBS 145572^T^	*Pinus sylvestris*	*Pinaceae*	Norway	MK876429	MK876388	MK876484	–
*D. gardeniae*	CBS 626.68^T^ = IMI 108771	*Gardenia jasminoides*	*Rubiaceae*	India	GQ387595	FJ427003	KT389606	FJ427114
*D. gei*	CGMCC 3.20068 = LC 13581^T^	*Geum* sp.	*Rosaceae*	China	MT229675	MT229698	MT239095	MT249266
*D. glomerata*	CBS 528.66 = PD 63/590	*Chrysanthemum* sp.	*Asteraceae*	The Netherlands	EU754184	FJ427013	GU371781	FJ427124
*D. gongkaensis*	YMF1.05095^T^	*Hippuris vulgaris*	*Hippuridaceae*	China	MH257458	MH257372	MH311812	MH422999
*D. gongkaensis*	YMF1.05029	*Hippuris vulgaris*	*Hippuridaceae*	China	MH257459	MH257373	MH311813	MH423000
*D. guttulata*	CBS 127976^T^	Soil		Zimbabwe	MN943730	MN973524	MT018138	MT005625
*D. heteroderae*	CBS 109.92^T^ = PD 73/1405	Undefined food material		The Netherlands	GU238002	FJ426983	KT389601	FJ427098
*D. hippuris*	YMF1.05089^T^	*Hippuris vulgaris*	*Hippuridaceae*	China	MH257473	MH257388	MH311827	MH423015
*D. hippuris*	YMF1.05204	*Myriophyllum spicatum*	*Haloragaceae*	China	MH257482	MH257397	MH311835	–
*D. ilicicola*	CGMCC 3.18355^T^ = LC 8126 = LC 8127	*Ilex chinensis*	*Aquifoliaceae*	Italy	KY742219	KY742065	KY742150	KY742307
*D. indica*	CBS 653.77^T^	Unknown		India	MN943741	MN973534	MT018159	MT005637
*D. infuscatispora*	CGMCC 3.18356^T^ = LC 8128	*Chrysanthemum indicum*	*Asteraceae*	China	KY742221	KY742067	KY742152	KY742309
*D. keratinophila*	CBS 143032^T^	Human superficial tissue		USA	LN907343	LT592901	LT593039	LT592970
*D. kooimaniorum*	CBS 144951^T^	Soil		The Netherlands	MN823299	MN823448	MN824474	MN824622
*D. lethalis*	CBS 103.25	Unknown	Unknown	Unknown	GU238010	GU237729	KT389607	GU237564
*D. ligulariae*	CGMCC 3.20070 = LC 13583^T^	*Ligularia sibirica*	*Asteraceae*	China	MT229676	MT229699	MT239096	MT249267
*D. longicolla*	CBS 124,514 = PD 80/1189^T^	*Opuntia* sp.	*Cactaceae*	Spain	GU238095	GU237767	MT018161	GU237622
*D. macrophylla*	CGMCC 3.18357 = LC 8131^T^	*Hydrangea macrophylla*	*Saxifragaceae*	Italy	KY742224	KY742070	KY742154	KY742312
*D. magnoliae*	MFLUCC 18-1560^T^	*Magnolia grandiflora*	*Magnoliaceae*	China	MK348033	MK347814	MK434852	–
*D. macrostoma*	CBS 223.69	*Acer pseudoplatanus*	*Aceraceae*	Switzerland	GU238096	GU237801	KT389608	GU237623
*D. maydis*	CBS 588.69^T^	*Zea mays*	*Poaceae*	USA	EU754192	FJ427086	GU371782	FJ427190
*D. microchlamydospora*	CBS 105.95^T^	*Eucalyptus* sp.	*Myrtaceae*	UK	GU238104	FJ427028	KP330424	FJ427138
*D. mitis*	CBS 443.72^T^	Soil		South Africa	MN943729	MN973523	MT018137	MT005624
*D. molleriana*	CBS 229.79 = LEV 7660	*Digitalis purpurea*	*Scrophulariaceae*	New Zealand	GU238067	GU237802	KP330418	GU237605
*D. musae*	CBS 463.69	*Mangifera indica*	*Anacardiaceae*	India	GU238011	FJ427026	MT018148	FJ427136
*D. myriophyllana*	YMF1.05035	*Myriophyllum aquaticum*	*Haloragaceae*	China	MH257484	MH257399	MH311837	MH423001
*D. myriophyllana*	YMF1.05100^T^	*Myriophyllum aquaticum*	*Haloragaceae*	China	MH257486	MH257401	MH311839	MH423003
*D. naikii*	PLS3^T^	*Cajanus cajan*	*Fabaceae*	India	OM830704	OM952211	–	OM858681
*D. negriana*	CBS 358.71	*Vitis vinifera*	*Vitaceae*	Germany	GU238116	GU237838	KT389610	GU237635
*D. nigricans*	PDDCC 6546 = CBS 444.81^T^	*Actinidia chinensis*	*Actinidiaceae*	New Zealand	GU238000	GU237867	MT018146	GU237558
*D. ocimicola*	CGMCC 3.18358^T^ = LC 8137	*Ocimum* sp.	*Lamiaceae*	China	KY742232	KY742078	MT018181	KY742320
*D. pedeiae*	PD 92/612A = CBS 124517^T^	*Schefflera elegantissima*	*Araliaceae*	The Netherlands	GU238127	GU237770	KT389612	GU237642
*D. pinodella*	CBS 531.66	*Trifolium pretense*	*Fabeceae*	USA	GU238017	FJ427052	KT389613	FJ427162
*D. pinodes*	CBS 525.77^T^	*Pisum sativum*	*Fabeceae*	Belgium	GU238023	GU237883	KT389614	GU237572
***D. pittospori***	**HJAUP C1740** ^T^	***Pittosporum tobira***	***Pittosporaceae***	**China**	**OR625711**	**OR625710**	–	**OR620205**
***D. pittospori***	**HJAUP C1800**	***Eriobotrya japonica***	***Rosaceae***	**China**	**OR905581**	**OR905550**	**OR947922**	**OR934715**
*D. pomorum*	CBS 539.66 = ATCC 16,791 = IMI 122,266 = PD 64/914	*Polygonum tataricum*	*Polygonaceae*	The Netherlands	GU238028	FJ427056	KT389618	FJ427166
*D. prolaticolla*	CBS 126182^T^	Surface soil		Namibia	MN943740	MN973533	MT018157	MT005636
*D. prosopidis*	CBS 136414^T^	*Prosopis* sp.	*Fabaceae*	South Africa	KF777232	KF777180	MT018149	MT005631
*D. protuberans*	CBS 381.96^T^ = PD 71/706	*Lycium halifolium*	*Solanaceae*	The Netherlands	GU238029	GU237853	KT389620	GU237574
*D. pteridis*	CBS 379.96^T^	*Pteris* sp.	*Pteridaceae*	The Netherlands	KT389722	KT389504	KT389624	KT389801
*D. qilianensis*	LC 13584	*Rheum officinale*	*Polygonaceae*	China	MT229677	MT229700	MT239097	MT249268
*D. qilianensis*	CGMCC 3.20071 = LC 13585^T^	*Rheum officinale*	*Polygonaceae*	China	MT229678	MT229701	MT239098	MT249269
*D. rhei*	CBS 109,177 = LEV 15,165 = PD 2000/9941	*Rheum rhaponticum*	*Polygonaceae*	New Zealand	GU238139	GU237743	KP330428	GU237653
*D. rumicicola*	CBS 683.79^T^ = LEV 15094	*Rumex obtusifolius*	*Polygonaceae*	New Zealand	KT389721	KT389503	KT389622	KT389800
*D. sancta*	CBS 281.83^T^	*Ailanthus altissima*	*Simaroubaceae*	South Africa	GU238030	FJ427063	KT389623	FJ427170
*D. segeticola*	CGMCC 3.17489^T^ = LC 1636	*Cirsium segetum*	*Asteraceae*	China	KP330455	KP330443	KP330414	KP330399
*D. senecionicola*	CBS 160.78 = LEV 11451	*Senecio jacobaea*	*Asteraceae*	New Zealand	GU238143	GU237787	MT018177	GU237657
*D. sinensis*	CGMCC 3.18348^T^ = LC 5210	*Cerasus pseudocerasus*	*Rosaceae*	China	KY742239	KY742085	MT018127	KY742327
*D. subglobispora*	CBS 364.91^T^	*Ananas sativus*	*Bromeliaceae*	Unknown	MN943737	MN973531	MT018153	MT005634
*D. subglomerata*	CBS 110.92 = PD 76/1010	*Triticum* sp.	*Poaceae*	USA	GU238032	FJ427080	KT389626	FJ427186
*D. subherbarum*	CBS 250.92^T^ = DAOM 171,914 = PD 92/371	*Zea mays*	*Poaceae*	Canada	GU238145	GU237809	MT018162	GU237659
*D. subrosea*	CBS 733.79^T^	*Abies alba litter*	*Pinaceae*	France	MN943747	MN973540	MT018174	MT005643
*D. suiyangensis*	CGMCC 3.18352^T^ = LC 7439	Air		China	KY742243	KY742089	KY742168	KY742330
*D. tabebuiicola*	COAD 3340^T^	*Tabebuia aurea*	*Bignoniaceae*	Brazil	MZ703623	MZ703618	MZ712360	MZ712364
*D. uniseptata*	CGMCC 3.20069 = LC 13582^T^	*Syringa vulgaris*	*Oleaceae*	China	MT229679	MT229702	MT239099	MT249270
*D. variabilis*	CBS 254.79^T^	*Vitis vinifera*	*Vitaceae*	Italy	MN943751	MN973544	MT018182	MT005647
*D. viburnicola*	CBS 523.73 = PD 69/800	*Viburnum cassioides*	*Adoxaceae*	The Netherlands	GU238155	GU237879	KP330430	GU237667
*Epicoccum camelliae*	CGMCC 3.18343 ^T^ = LC 4858	*Camellia sinensis*	*Theaceae*	China	KY742245	KY742091	KY742170	KY742333
*E. camelliae*	LC 4862	*Camellia sinensis*	*Theaceae*	China	KY742246	KY742092	KY742171	KY742334
*E. latusicollum*	CGMCC 3.18346^T^ = LC 5158	*Sorghum bicolor*	*Poaceae*	China	KY742255	KY742101	KY742174	KY742343
*E. latusicollum*	LC 4859	*Camellia sinensis*	*Theaceae*	China	KY742256	KY742102	KY742175	KY742344
*E. latusicollum*	LC 5124	*Vitex negundo*	*Lamiaceae*	China	KY742257	KY742103	–	KY742345
*E. nigrum*	CBS 173.73^T^ = ATCC 24,428 = IMI 164070	*Dactylis glomerata*	*Poaceae*	USA	GU237975	FJ426996	KT389632	FJ427107
*E. poae*	CGMCC 3.18363^T^ = LC 8160	*Poa annua*	*Poaceae*	USA	KY742267	KY742113	KY742182	KY742355
*E. sorghinum*	CBS 179.80 = PD 76/1018	*Sorghum vulgare*	*Poaceae*	PuertoRico	GU237978	FJ427067	KT389635	FJ427173
*E. sorghinum*	CBS 627.68 = PD 66/926	*Citrus* sp.	*Rutaceae*	France	GU237979	FJ427072	KT389636	FJ427178
*Paraboeremia adianticola*	CBS 187.83 = PD 82/128	*Polystichum adiantiforme*	*Dryopteridaceae*	USA	GU238035	GU237796	KP330401	GU237576
*P. adianticola*	CBS 260.92 = PD 86/1103	*Pteris ensiformis*	*Pteridaceae*	–	KT389752	KT389534	–	KT389832
*P. camellae*	CGMCC 3.18106^T^ = LC 4852	*Camellia* sp.	*Theaceae*	China	KX829042	KX829034	KX829050	KX829058
*P. camellae*	CGMCC 3.18107 = LC 6253	*Camellia* sp.	*Theaceae*	China	KX829043	KX829035	KX829051	KX829059
*P. oligotrophica*	CGMCC 3.18111^T^ = LC 6250	Carbonatite		China	KX829039	KX829031	KX829047	KX829055
*P. oligotrophica*	CGMCC 3.18112 = LC 6251	Carbonatite		China	KX829040	KX829032	KX829048	KX829056

Notes: “–”, sequence is unavailable. Strain with T (ex-type). Abbreviations: **ATCC:** American Type Culture Collection, Virginia, U.S.A.; **CBS:** Westerdijk Fungal Biodiversity Institute (formerly CBSKNAW), Utrecht, The Netherlands; **CGMCC:** China General Microbiological Culture Collection, Beijing, China; **CREADC:** Consiglio per la Ricerca in Agricoltura e l’analisi dell’economia agraria, Centro di ricercarper la Difesa e la Certificazione, Roma, Italy; **DAOM:** Canadian Collection of Fungal Cultures, Ottawa, Canada; **DSM:** Deutsche Sammlung von Mikroorganismen und Zellkulturen GmbH, Braunschweig, Germany; **FMR:** Faculty of Medicineand Health Sciences culture collection, Reus; **HJAUP:** Herbarium of Jiangxi Agricultural University, Plant Pathology; **IMI:** International Mycological Institute, CABI-Bioscience, Egham, Bakeham Lane, U.K.; **IRAN:** Iranian Fungal Culture Collection, Iranian Research Institute of Plant Protection, Tehran, Iran; **LC:** Corresponding author’s personal collection deposited in laboratory, housed at CAS, China; **LEV:** Plant Health and Diagnostic Station, Auckland, New Zealand; **MFLUCC:** Mae Fah Luang University Culture Collection; **PD:** Plant Protection Service, Wageningen, The Netherlands; **PDDCC:** Plant Diseases Division Culture Collection, Auckland, New Zealand; **PREM:** National Collection of Fungi: Culture Collection, Pretoria, South Africa; **YMF:** Herbarium of the Laboratory for Conservation and Utilization of Bio-Resources, Yunnan University, Kunming, Yunnan, China; **ITS:** internal transcribed spacer; **LSU:** large subunit ribosomal; ***RPB2*:** second largest subunit of RNA polymerase II; ***TUB2*:** β-tubulin.

## Data Availability

All sequences generated in this study were submitted to GenBank.
